# Endoscopic mucoplasty as a novel approach for cervical esophageal web

**DOI:** 10.1055/a-2686-3762

**Published:** 2025-09-09

**Authors:** Kazuki Yamamoto, Haruhiro Inoue, Yohei Nishikawa, Kei Ushikubo, Kohei Shigeta, Ippei Tanaka, Mayo Tanabe

**Affiliations:** 1 378609 Digestive Disease Center, Showa Medical University Koto Toyosu Hospital, Tokyo, Japan

Esophageal webs are thin, membranous structures, most commonly found in the cervical esophagus, that can obstruct the lumen and cause dysphagia 1 . While balloon dilation can be a common treatment for esophageal webs, the cervical esophagus presents anatomical challenges and higher risks, making repeated dilation less favorable. We previously reported the utility of endoscopic mucoplasty (EMP) in treating symptomatic, refractory Schatzki rings 2 . Building on this, we present a case where EMP successfully treated a cervical esophageal web.

A 39-year-old man was referred with persistent dysphagia. Initial esophagogastroduodenoscopy (EGD; GIF-1200N, Olympus) could not pass through the cervical esophagus due to a thin membranous stricture ( Fig. 1 **a** ). Barium swallow revealed a 3- to 4-cm concentric narrowing ( Fig. 1 **b** ). Laboratory tests ruled out iron deficiency anemia and autoimmune diseases 3 , and contrast-enhanced CT showed no mass lesions or external compression.

**Fig. 1:**
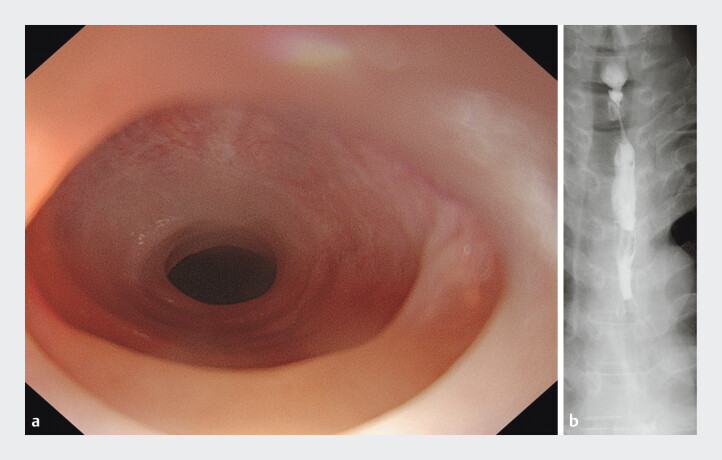
**a** Initial esophagogastroduodenoscopy (EGD; GIF-1200N, Olympus) is unable to pass through the cervical esophagus due to a thin membranous stricture. **b** A barium esophagram shows a 3- to 4-cm segment of concentric narrowing in the cervical esophagus.

EMP was performed using an EG-840TP endoscope ( Video 1 ). A submucosal tunnel was created proximal to the stricture with a Triangle Tip Knife J (Olympus) and an electrosurgical unit (VIO3; ERBE, Endocut I: 1-3-3) ( Fig. 2 **a–d** ). A longitudinal incision was made from the tunnel entry to the distal end of the stricture. The procedure was completed after confirming adequate luminal expansion and smooth endoscope passage ( Fig. 3 **a–d** ).

Endoscopic mucoplasty for cervical esophageal web.Video 1

**Fig. 2:**
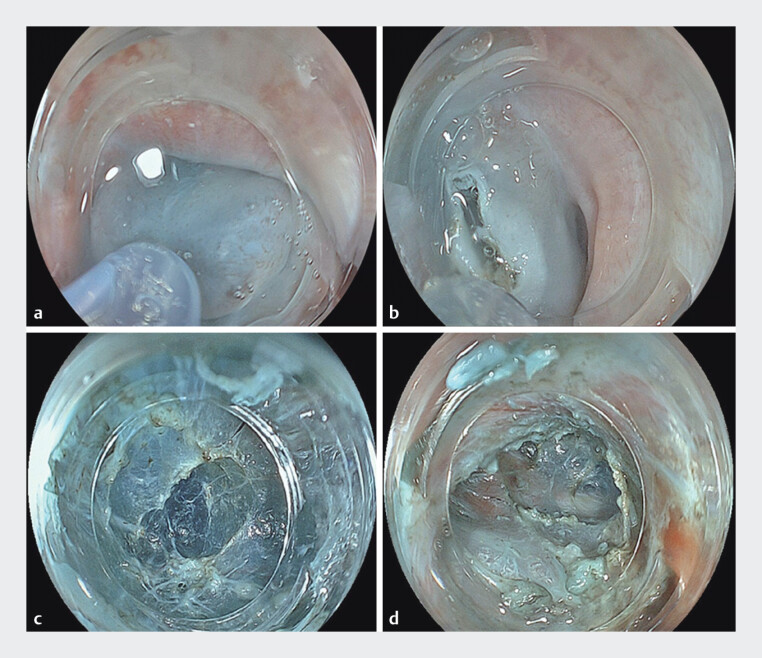
**a** Submucosal injection of saline mixed with indigo carmine is performed using a 25-G, 4-mm tip needle (NeedleMaster; Olympus). **b** A mucosal incision is made to access the submucosal space using a Triangle Tip Knife J (Olympus). **c** The submucosal layer is carefully dissected to create a submucosal tunnel. **d** A submucosal tunnel approximately 3–4 cm in length is created.

**Fig. 3:**
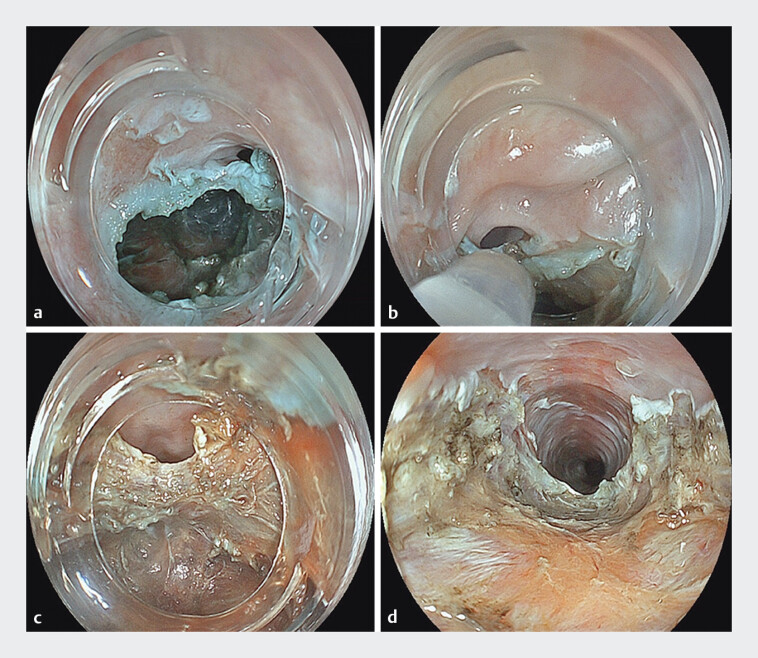
**a** Endoscopic view of the submucosal tunnel and esophageal lumen before the longitudinal mucosal incision. **b** A longitudinal mucosal incision is initiated from the entry point of the tunnel. **c** The incision is extended distally to the far end of the stricture. **d** The procedure is completed after confirming sufficient luminal expansion and smooth endoscope passage.

The patient recovered uneventfully, began clear liquids on postoperative day one, and gradually resumed a regular diet. Symptoms resolved promptly, and he was discharged on day four. At one-month follow-up, he remained asymptomatic. Surveillance EGD (GIF-1200N, Olympus) showed no restenosis, and the endoscope could traverse the segment without resistance ( Fig. 4 **a** ). Barium swallow confirmed smooth esophageal passage ( Fig. 4 **b** ). High-resolution manometry ( Fig. 5 ) and random biopsies showed no abnormalities, and the web was diagnosed as idiopathic.

**Fig. 4:**
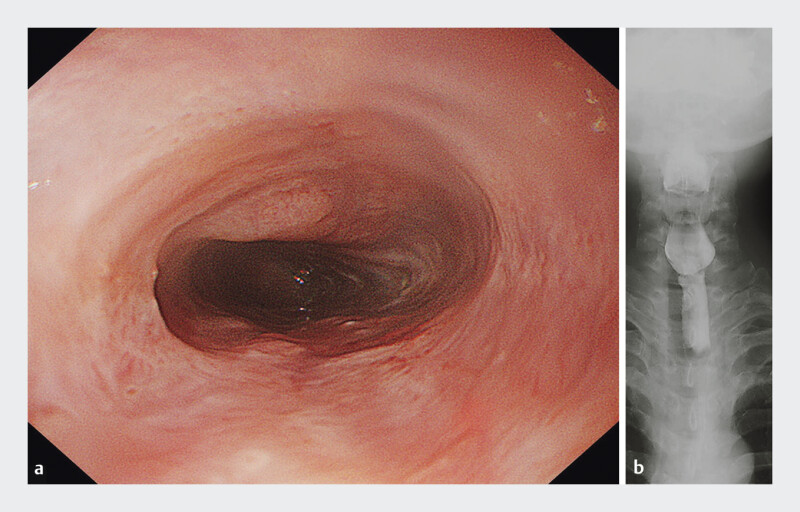
**a** Follow-up esophagogastroduodenoscopy (EGD; GIF-1200N, Olympus) at one month demonstrates sustained luminal patency, with the endoscope easily traversing the previously narrowed cervical segment. **b** A barium esophagram performed at the same interval confirms unobstructed and smooth passage through the cervical esophagus.

**Fig. 5:**
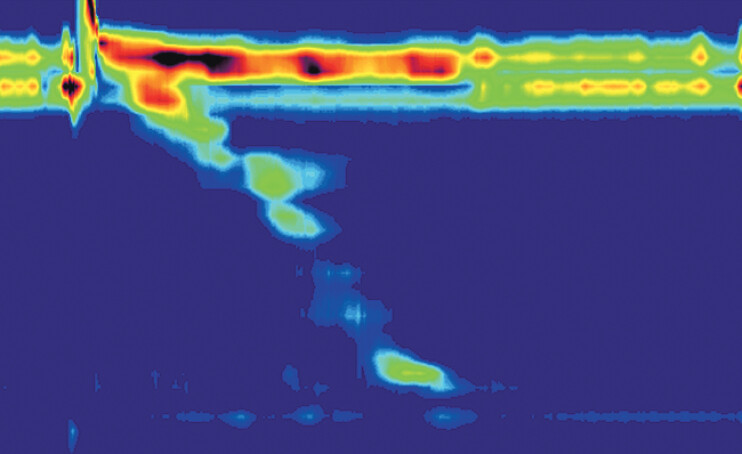
High-resolution esophageal manometry reveals normal esophageal motility with no signs of lower esophageal sphincter dysfunction. The integrated relaxation pressure (IRP) averages 6.6 mmHg, distal latency (DL) is measured at 9.6 seconds, and the peak distal contractile integral (DCI) is 318.1 mmHg s cm.

This case highlights EMP as a safe, effective alternative, even in anatomically challenging locations beyond Schatzki ring management.

Endoscopy_UCTN_Code_TTT_1AO_2AH

## Notes

Endoscopy E-Videos https://eref.thieme.de/e-videos
*E-Videos* is an open access online section of the journal *Endoscopy* , reporting on interesting cases and new techniques in gastroenterological endoscopy. All papers include a high-quality video and are published with a Creative Commons CC-BY license. Endoscopy E-Videos qualify for HINARI discounts and waivers and eligibility is automatically checked during the submission process. We grant 100% waivers to articles whose corresponding authors are based in Group A countries and 50% waivers to those who are based in Group B countries as classified by Research4Life (see: https://www.research4life.org/access/eligibility/ ). This section has its own submission website at https://mc.manuscriptcentral.com/e-videos .
